# Task demands shape network interactions during reading and visual form processing

**DOI:** 10.1162/IMAG.a.13

**Published:** 2025-05-28

**Authors:** Vicky He, Bahman Tahayori, David N. Vaughan, Heath R. Pardoe, Jodie E. Chapman, Graeme D. Jackson, David F. Abbott, Chris Tailby, for the Australian Epilepsy Project Investigators

**Affiliations:** The Florey Institute of Neuroscience and Mental Health, Heidelberg, Victoria, Australia; Florey Department of Neuroscience and Mental Health, The University of Melbourne, Parkville, Victoria, Australia; Department of Neurology, Austin Health, Heidelberg, Victoria, Australia; Department of Medicine—Austin Health, The University of Melbourne, Heidelberg, Victoria, Australia; Department of Clinical Neuropsychology, Austin Health, Heidelberg, Victoria, Australia

**Keywords:** fMRI, psychophysiological interaction, functional connectivity, dynamic brain networks, reading, language

## Abstract

Current theories of reading, based largely on lesion studies, propose that left fusiform gyrus (FusG) is a crucial hub linking visual inputs into the perisylvian language system. However, FusG is also involved in visual form processing more generally, raising the question of whether its engagement with language cortex and other relevant brain regions changes according to task demands, and whether this flexibility is linked to reading proficiency. To answer these questions, we exploit a large, pre-existing, reading-based language fMRI dataset, in which pseudoword rhyming and visuospatial form processing are contrasted. We studied 201 adults from the Australian Epilepsy Project, including 94 with a history of seizures. A comparable pattern of activation and connectivity was observed in those with or without a history of seizures: The rhyming task produced robust activation in left FusG and perisylvian language areas. As predicted, connectivity from FusG to the left language cortex increased significantly during rhyming. We also observed significant increases in connectivity from the same FusG seed to the right parietal cortex during visuospatial form processing. The seizure group comprised a broader range of reading proficiency, and in this group better readers showed stronger increases in FusG connectivity to the left middle frontal gyrus and the medial prefrontal cortex. This suggests that visual text processing and articulatory sequencing are less tightly integrated in poor readers. By demonstrating how FusG connectivity shifts between language and visuospatial networks, our results provide new evidence that adaptive functional reorganisation is beneficial for cognitive performance.

## 1 Introduction

There are well-developed neurocognitive models of reading connecting visual processing networks with perisylvian language networks (Price, 2012;Sandak et al., 2004). Prior research in healthy volunteers and in individuals with developmental and acquired reading problems has converged on a stereotyped sequence of neural activity underpinning reading, especially reading of pseudowords (e.g., “glat” or “frung”) which contain phonological but not semantic information. This process starts with the relay of visual information to primary visual cortices. From there, the information is transmitted and processed along pathways through the posterior lateral bank of the left fusiform gyrus (FusG), sometimes referred to as the Visual Word Form Area (Goodale & Milner, 1992;Price, 2012;Sandak et al., 2004). FusG is involved in identifying letters, combinations of letters, and words from lower-level shape images, acting as a gateway between vision and language (Baker et al., 2007;Dehaene & Cohen, 2007;Hillis et al., 2005). Once a word-related visual feature has been processed in FusG, it must be integrated with perisylvian language processing regions such as left superior temporal sulcus and gyrus (STS and STG, respectively; proximal to so-called Wernicke’s area) and left inferior frontal gyrus (IFG; proximal to Broca’s area;Yarkoni et al., 2008). Left posterior STS and STG are implicated in phonological processing (Price, 2012), and left IFG in auditory-articulatory mapping (Price, 2012;Sandak et al., 2004).

It is unlikely that the FusG is involved exclusively with reading (Dehaene & Cohen, 2007,^2011^). The FusG is situated along the ventral visual pathway concerned with form analysis (Goodale & Milner, 1992) and exhibits features important for visual form processing more generally (Chen et al., 2019;Price & Devlin, 2003;Vogel et al., 2014). Viewed in this light, the contribution of FusG to reading is but one example of the contribution of this region to visual form processing more broadly. This idea is captured in the multiplex model of FusG outlined by Chen and colleagues on the basis of resting state and diffusion imaging (Chen et al., 2019).

The multiplex model proposes that a single brain region, such as the FusG, can be recruited into different networks to perform various tasks. A key testable hypothesis is that connectivity between regions varies systematically in response to changing task demands. In other words, multiplexing predicts that task state modulates the degree to which moment-to-moment fluctuations in activity covary between brain areas (over and above any changes in mean signal across those regions, as captured by standard ‘localisation of activation’ analyses). Here, we test this multiplexing idea (Chen et al., 2019) explicitly by examining the hypothesis that connectivity from FusG to perisylvian language cortex is modulated according to linguistic demands. More specifically, if an attended visual input is to undergo grapheme-to-phoneme conversion as in pseudoword reading, there will be an increase in communication between FusG and perisylvian language regions relative to when handling comparable non-language related visual input. Furthermore, we hypothesize that the magnitude of this state-dependent change in connectivity from FusG to language cortex is associated with overall reading proficiency. That is, compromise of the hypothesized multiplexing property of FusG would be associated with reading difficulty.

We tested these hypotheses by leveraging an existing large-scale dataset from the Australian Epilepsy Project (AEP;https://www.epilepsyproject.org.au), an ongoing study recruiting individuals with seizure disorders and healthy volunteers. The project contains functional magnetic resonance imaging (fMRI) data from a language rhyming task and a visuospatial pattern matching task, along with behavioral measurements, such as reading abilities, making it suitable for examining the multiplexing hypothesis. In addition, the AEP benefits from sampling across a diverse range of reading abilities, as reading difficulties are relatively common in epilepsy (Lah et al., 2017;Tailby et al., 2014). The AEP had recruited over 200 participants at the time of writing, all capable of reading in English.

**Fig. 1.**
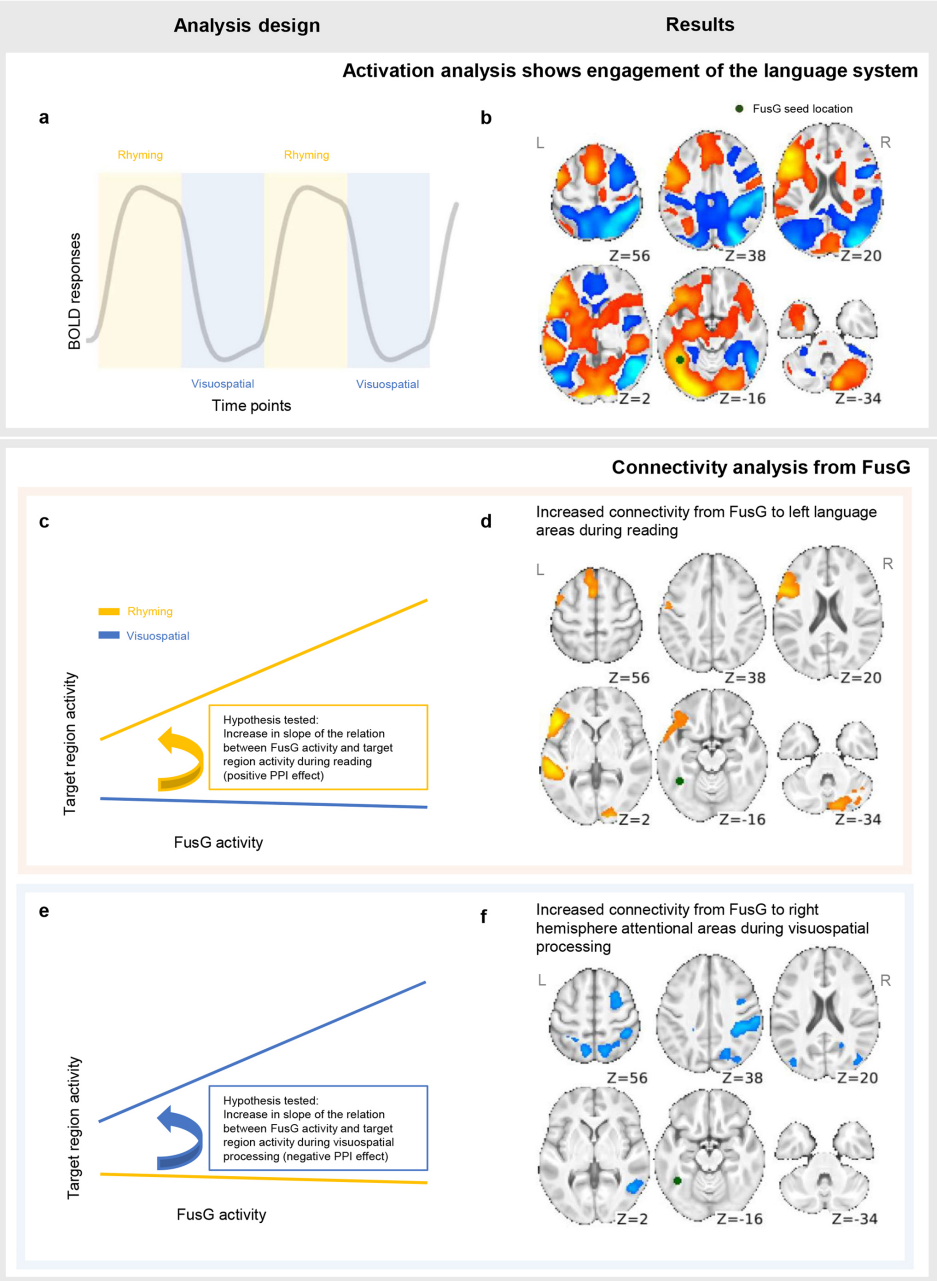
Pseudoword rhyming activation and FusG task modulated connectivity analyses. (a, b) Task-related activation was estimated by fitting the BOLD response at each voxel with a box car convolved with the canonical HRF, as schematically illustrated in (a), then combined across all 201 participants using a one sample t-test to yield a statistical parametric map of activation during pseudoword rhyming (b). This shows strong left lateralised activation in classical language areas, and strong right lateralised deactivation in frontoparietal and lateral posterior temporal cortices. (c–f) Task-dependent changes in functional connectivity were estimated using PPI analysis, which models changes in the slope of the relation between activity in a seed region, FusG (green sphere in slice Z = -16 in b, d, and f), and activity in other brain areas as a function of task state (illustrated schematically in c and e). This change in connectivity/slope is captured by the PPI parameter in the multiple regression model (see Methods). As modeled here, increases in connectivity between FusG and other brain areas during rhyming will result in positive PPI parameter estimates (c), whereas increases in connectivity during visuospatial matching will result in negative PPI parameter estimates (e). One-sample*t*-test on the PPI parameters across all participants reveals that connectivity from FusG increased to left perisylvian language cortex, left superior medial frontal cortex, and right cerebellum during rhyming (d), but that connectivity from FusG increased to right frontoparietal and lateral temporal cortex during visuospatial matching (f). All SPM-t maps were generated using a feature inducing threshold of*p*< 0.001 uncorrected, FWEc*p*< 0.05 (two-tailed); the left hemisphere is shown on the left.

We analyzed the fMRI data via psychophysiological interaction (PPI) analysis (Friston et al., 1997), a multiple regression method that captures both task-dependent activation and deactivation (Fig. 1a), as used in conventional fMRI analyses, as well as task-dependent changes in the functional connectivity from a seed region (here, FusG) to other brain areas (Fig. 1c, e).

## 2 Methods

### 2.1 Participants

The study was conducted with two groups of consecutively recruited participants from the AEP: 124 participants with seizure disorders and 114 healthy controls. AEP participants are adults aged between 18 and 67 years, required to have a functional level of English; exclusion criteria are a moderate or severe intellectual disability and/or contraindications for 3T MRI. The study was approved by the Austin Health Human Research Ethics Committee (HREC/60011/Austin-2019 and HREC/68372/Austin-2022).

From our initial sample of 124 participants with seizure disorders, we excluded 22 whose MRI scans could not be used in group-level analyses: 1 with excessive motion artefacts (defined inSection 2.5), 21 with gross structural abnormalities or previous surgeries, and 8 with non-left language lateralization (i.e., the dominant hemisphere activated by the language task is not on the left). Language lateralization was categorized based on methods described inAbbott et al. (2010). The final number of participants in the seizure group included in the analyses is 94, drawn from three target seizure cohorts per AEP inclusion criteria (Tailby et al., 2020): 26 with a first unprovoked seizure (FUS), 29 with newly diagnosed epilepsy (NDE), and 39 with drug-resistant epilepsy (DRE). None of the FUS participants were on antiseizure medications (ASM). Medication data were available for 15 of 29 NDE and 19 of 39 DRE participants, with NDE participants taking a median of 1 ASM and DRE participants taking a median of 2 ASMs. Among controls, we excluded 3 participants due to image artefacts, and 4 due to non-left lateralised language activation. The final number of healthy controls included in the analyses was 107. Demographic information of the final sample is provided inTable 1. Median ages (in years) of the seizure and control groups were 33 and 43, respectively (*p*< 0.001, Mann-Whitney-Wilcoxon test due to non-normal distribution). There were 47 (50%) males in the seizure group and 38 (36%) in controls (*p*= 0.05, chi-square test). Controls had more years of education and higher IQ than the seizure group (both*p*< 0.001, Mann-Whitney-Wilcoxon tests).

**Table 1.** Participant characteristics.

	Seizure ( *N* = 94)	Control ( *N* = 107)		
	Median or count	Median or count		
Variable	(IQR or %)	(IQR or %)	Test Statistics	*p* -value
Age (years)	33 (19.5)	43 (22.0)	W = 3395	<0.001
Males	47 (50.0)	38 (35.5)	Chi-square = 3.73	0.053
Reading scores	102 (22.0)	111 (12.8)	W = 2822	<0.001
Education (years)	15 (3.0)	15 (3.0)	W = 3262	<0.001
IQ	101 (19.0)	110 (17.5)	W = 2694	<0.001

Target seizure cohorts				
FUS	26 (27.7)			
NDE	29 (30.9)			
DRE	39 (41.5)			
Epileptogenic abnormality				
Yes	17 (18.1)			
No	59 (62.8)			
Equivocal	18 (19.1)			

Age, reading scores, education in years, and IQ are given in median (interquartile range, IQR); sex (number of males), target seizure cohorts of patients, and whether there was epileptogenic abnormality are given as count (%).

FUS: first unprovoked seizure; NDE: newly diagnosed epilepsy; DRE: drug-resistant epilepsy.

### 2.2 Reading scores

All participants completed the Test of Premorbid Functioning (ToPF;Wechsler, 2011), a measure based on the pronunciation of irregular words. The ToPF provides an estimate of reading ability and is related to vocabulary set size. Raw scores were converted to age-adjusted standard scores. We refer to ToPF scores as reading scores in this manuscript. Reading scores were available for 89 of the 94 participants in the seizure group. There were 2 seizure participants with missing reading scores, and 3 with invalidated data due to English not being their first language. Reading scores were available for 94 of the 107 controls, with 70 controls having valid scores, 24 having imputed scores, and 13 having invalid scores. The imputation was performed using data from separate work, where a cohort of 272 individuals completed both the ToPF and a locally developed reading metric. We fitted a linear regression model predicting ToPF scores from the local reading metric in this group (*r*= 0.96,*p*< 0.001) and used the resulting regression equation to impute ToPF scores for the 24 cases in the present study where a full ToPF score was not available. Reading scores were lower in the seizure group (*p*< 0.001, Mann-Whitney-Wilcoxon test due to non-normal distribution; see alsoFig. 2b). Within the seizure group, reading scores did not differ between target seizure cohorts (*p*= 0.3, Kruskal-Wallis rank sum test due to non-normal distribution).

**Fig. 2.**
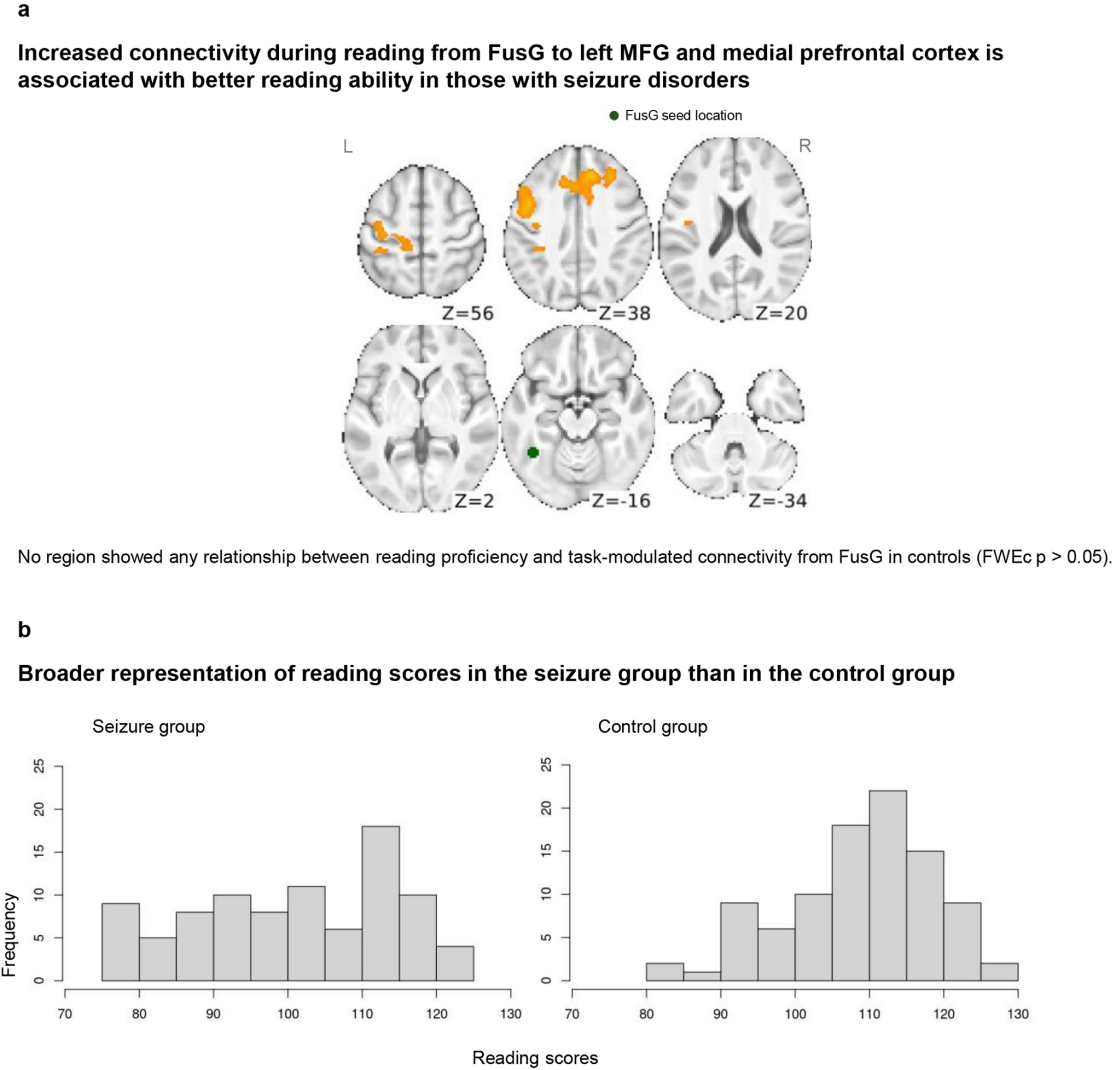
(a) In the seizure group, better reading ability is associated with stronger task-modulated connectivity from FusG to left MFG and to bilateral medial frontal cortex. FusG seed location is shown in green (slice Z = -16). Left hemisphere on the left side. FWEc*p*< 0.05 (two-tailed). (b) Distribution of reading scores in the seizure group and the control group. The seizure group had a broad distribution of reading scores, whereas the control group had an under-representation of low reading scores.

### 2.3 MRI data acquisition and preprocessing

We collected T1-weighted as well as Multi-Band Multi-Echo (MBME) fMRI images for all AEP participants performing a language task (details below) in a 3T Siemens PrismaFit MRI scanner, with the following parameters: three echoes at TE = [15 33.25 51.5] ms, TR = 0.9 s, multi-band factor = 4, GRAPPA factor = 2, Field of View (FOV) = 216 × 216 × 132 mm^3^, Flip Angle (FA) = 30^◦^, bandwidth = 2670 Hz/px, and 200 volumes per subject with anterior-posterior phase encoding direction. Phase-encode-reversed pairs of spin-echo images were acquired to enable adjustment for susceptibility distortion on the fMRI data. T1-weighted MPRAGE images with TE = 2.19 ms, TI = 1 s, TR = 1.9 s, FA = 8^◦^as well as T2-weighted FLAIR images with TE = 392 ms, TI = 1.8 s, TR = 5 s, FA = 120^◦^were acquired. For both types of structural images, the voxel size, FOV, and GRAPPA factor were 3 × 3 × 3 mm^3^, 230 × 230 × 173 mm^3^, and 2, respectively. Image preprocessing included using MRtrix3 software version 3.0.4 (Tournier et al., 2019) to apply Marchenko-Pastur PCA (MPPCA) denoising (Veraart et al., 2016), which can help remove random thermal noise in fMRI (Ades-Aron et al., 2021;Mosso et al., 2022). This was followed by fMRIPrep 21.0.2 (Esteban et al., 2019,^2020^). Functional images were smoothed with an 8 mm full width at half maximum Gaussian kernel. Further details are provided inSupplementary Methods.

### 2.4 fMRI paradigm: pseudoword rhyming

All participants completed a language fMRI task (Tailby et al., 2017,^2020^). This is a block design task, alternating task-active and baseline phases of 18 s duration. In the task-active phase, participants decide whether visually presented pairs of pseudowords rhyme or not (e.g., “blint” vs. “glat”); participants completed the task silently (i.e., without speaking aloud) and it requires grapheme-to-phoneme conversion. Using pseudowords, rather than real words, has the advantage of tapping grapheme-to-phoneme conversion directly without concurrent recruitment of semantics (Price, 2012). This provides a simplified interrogation of the interactions between the occipitotemporal reading system and classical perisylvian speech processing areas. In the baseline phase, participants decide whether pairs of line patterns consisting of forward and backward slashes are identical (e.g., “//\\” vs. “\\\/”), a visuospatial processing task. Each visual stimulus pair (rhyming and pattern) was shown for 4.5 s. The fMRI experiment began with two pattern matching pairs, then alternating blocks of four rhyming pairs and four pattern matching pairs until five blocks of the rhyming tasks were completed. This was followed by two more pattern matching pairs, yielding a total of 20 rhyming pairs and 20 pattern pairs. The total experiment duration was 180 s (200 TRs).

### 2.5 Task-activation analysis

FusG was localized by analyzing for activation during pseudoword rhyming. First-level analysis was performed using the iBT software version 3.9 (Abbott et al., 2024) with SPM12 (Penny et al., 2006) revision 7771. We discarded the first 10 volumes (9 s, the 2 pattern matchings) to allow the participants to settle. The total number of included volumes is 190. A general linear model (GLM) was used to contrast activation between the pseudoword rhyming task (coded as 1) and the pattern matching baseline (coded as 0). A box-car function convolved with canonical haemodynamic response function (HRF) was used to model task activation. We used framewise displacement to define scan-to-scan motion. When gross motion occurs (scan-to-scan motion greater than 1 mm), we rejected the initial motion-affected volume and the three subsequent volumes by including scan-nulling regressors. If scan-nulling regressors exceeded 50% of task or rest blocks, the participant was excluded from subsequent analysis. Twenty-four head motion parameters (3 translation and 3 rotation head motion parameters, 6 parameters about their position in the previous scan, and the square of these 12 parameters;Friston et al., 1996), the first eigenvariate of the white matter and cerebrospinal fluid (CSF), and a constant term were included as regressors of no interest. The model included a high-pass filter with a cut-off of 128 s and pre-whitening with a FAST model, which is recommended for TR less than 1 s (Corbin et al., 2018;Olszowy et al., 2019). The contrast of interest coded task-minus-baseline. One sample*t*-tests were performed on all individual first-level estimates of the task activation in the seizure group (*n*= 94), controls (*n*= 107), and seizure and controls combined (*n*= 201). In addition, we ran a two-sample*t*-test to compare task activation maps of the seizure group and controls. A two-tailed Family-Wise-Error cluster corrected (FWEc) threshold of*p*< 0.05 was applied, with an initial cluster forming threshold of*p*< 0.001 uncorrected.

### 2.6 Task-modulated connectivity analysis

PPI analysis (Friston et al., 1997) was applied to estimate task-dependent connectivity from FusG in each group of participants. The PPI regression model estimates changes in functional connectivity between a seed and a target, under different task conditions. This method falls somewhere between sliding windows used to capture short time scales, and static functional connectivity used to describe resting state (or single task) connectivity. The regression includes main effect of the task, main effect of the seed time course, and the interaction between the two (the PPI regressor; seeFig. 1for a schematic illustration). Since the relevant interactions occur at the neuronal level rather than the level of the haemodynamic responses, it has been recommended to deconvolve the HRF from the seed time course to capture the interactions more accurately. Therefore, a suggested approach is to construct the interaction term by multiplying the task regressor with the deconvolved seed time course, followed by convolving their product with the HRF (Gitelman et al., 2003).

We used the gPPI toolbox (McLaren et al., 2012) version 13.1 with SPM12 to implement voxelwise PPI analysis for every participant. The toolbox incorporates deconvolution during creation of the PPI term. We defined the seed region using a sphere of 6 mm radius, centered on the peak activation coordinate in left FusG from the one sample random effect*t*-test (*n*= 201) modeling the task activation (MNI [-40 -50 -20], green sphere inFig. 1b). The peak coordinates are proximal to published coordinates for the Visual Word Form Area (Cohen et al., 2002;Vogel et al., 2014). To derive the seed time course for each participant, we extracted the first eigenvariate across all voxels within the spherical region of interest (ROI). The interaction term was calculated by mean-centering the task regressor prior to multiplication with the deconvolved seed time course, as recommended (Di et al., 2017). This mean-centering approach is not available within the current release version of the gPPI package (v13.1) at the time of writing; we, therefore, implemented it by modifying the relevant line of code [Line 705 in PPPI.m function, changing ‘PSYxn(:,j) = PSY(:,j).*xn’ to ‘PSYxn(:,j) = (PSY(:,j)-mean(PSY(:,j))).*xn’]. The PPI model also included the 24 head motion parameters, the first eigenvariate of the white matter and CSF, and a constant term as regressors of no interest. One contrast image capturing the beta estimates on the PPI effect (interaction) was generated for each participant. One-sample*t*-tests were performed on the PPI effects in the seizure group (*n*= 94), controls (*n*= 107), and seizure and controls combined (*n*= 201). In addition, we ran a two-sample*t*-test to compare PPI maps of the seizure group and controls. Both the positive and negative PPI effects were of interest, hence a two-tailed FWEc threshold of*p*< 0.05 was applied, with an initial cluster-forming threshold of*p*< 0.001 uncorrected.

### 2.7 Task-modulated connectivity and reading proficiency

We further ran a whole-brain mixed effect regression model to investigate whether the magnitude of task-modulated connectivity covaried with reading performance, and whether this relationship differed between the seizure group (*n*= 89) and controls (*n*= 94). The regression model takes individual PPI estimates as the outcome variable, and predictors include reading scores and group (coded 1 for seizure and 0 for controls). Inspection of an initial model dummy coding the three seizure cohorts (FUS, NDE, DRE) separately indicated no significant difference in the relationship between reading scores and PPI estimates between the cohorts (FWEc*p*> 0.05, two-sided), so we combined them into a single seizure group for the main regression model. We also included an interaction between reading scores and group. Age, sex, and a constant term were included as regressors of no interest. One-sample*t*-tests (*n*= 183) were then performed on the beta estimates of the group and reading scores predictors, as well as the interaction effect. A two-tailed FWEc threshold of*p*< 0.05 was applied, with an initial cluster-forming threshold of*p*< 0.001 uncorrected.

## 3 Results

### 3.1 Pseudoword rhyming elicits activation in a left lateralised language network

All participants completed a block design task, alternating periods where they judged whether visually presented pairs of pseudowords rhyme (e.g., “blint” vs. “glat”) with periods where they decided whether pairs of strings of forward and backward slashes were identical (e.g., “//\\” vs. “\\\/”). The task contrasts visual form processing for the extraction of phonology with visual form processing for purely visuospatial structure. One patient participant was excluded due to excessive motion (76 usable volumes left out of 190). All included participants had at least 118 usable volumes (median = 190). The mean accuracy on the rhyming task was 92% (89% in the seizure group and 95% in the control group), with a mean reaction time of 1684 ms (1843 ms in the seizure group and 1540 ms in controls). The mean accuracy on the pattern matching baseline was 97% (96% in the seizure group and 98% in controls), with a mean reaction time of 1387 ms (1426 ms in the seizure group and 1352 ms in controls). In both the seizure disorder and control groups, standard activation analysis based on one-sample*t*-test on the contrast of pseudoword rhyming versus pattern matching reveals a typical pattern of left hemisphere dominant language activation. As expected, there were no group differences between activation maps of the seizure group and controls, so we combined them into a single group (Fig. 1b; seeSupplementary Fig. S1for group specific results). This shows left predominant activation in classical language distribution encompassing the posterior frontal cortex, lateral temporal cortex, and FusG. There is also prominent deactivation in the right intraparietal sulcus, right posterior superior frontal sulcus (frontal eye field), and right posterior middle temporal gyrus.

### 3.2 Increased connectivity from FusG during pseudoword rhyming

Our principal hypothesis was that connectivity from FusG would increase to left frontal and temporal language areas during pseudoword rhyming relative to visuospatial form processing. We implemented a PPI model seeded from the rhyming activation maxima in left FusG (green sphere inFig. 1b; MNI [-40 -50 -20]) to investigate task-modulated connectivity from FusG. As hypothesized, there was robust evidence of changes in connectivity from FusG according to the cognitive demands of the task at hand (PPI effects). This effect was common across groups, so we report on the combined effects (seeSupplementary Fig. S1for group-specific results).Figure 1dshows increased connectivity (positive PPI estimates) from FusG to classical language regions, specifically left IFG (MNI [-48 26 -2]) and STG (MNI [-54 -34 2]), during pseudoword rhyming. In addition, we identified discrete suprathreshold clusters in right cerebellum Crus I and II (MNI [36 -64 -26]) and left superior frontal gyrus (SFG), encompassing left frontal pole, supplementary motor area (SMA), and precentral gyrus. Details of the clusters are given inSupplementary Table S1.

### 3.3 Increased connectivity from FusG to right parietal cortex during visuospatial judgements

In addition to the positive interactions from FusG to the language cortex during pseudoword rhyming, we observed significant negative interactions (negative PPI estimates) in right intraparietal sulcus (MNI [60 -26 42]), at the junction of the right superior frontal and precentral sulci (MNI [26 -6 50]; right frontal eye field), and left lateral occipital cortex (MNI [-42 -70 12]). These areas of negative interaction imply stronger connectivity with FusG during visuospatial pattern matching relative to pseudoword rhyming, providing additional evidence of task-dependent modulation of connectivity from FusG (Fig. 1f). Details of the clusters are given inSupplementary Table S2.

### 3.4 Reading proficiency associated with increased connectivity from FusG in seizure group

We next tested the hypothesis that stronger task-modulated connectivity from FusG would be associated with increased reading proficiency. We did not observe common effects across groups so report them separately here. As shown inFigure 2a, we found positive linear relationships between reading proficiency and task-modulated connectivity from FusG in left middle frontal gyrus (MFG; MNI [-44 10 36]) extending to left precentral and postcentral gyri (MNI [-34 -34 46]), as well as bilateral medial prefrontal/paracingulate cortices (MNI [14 34 36]) in the seizure group. In the control group, we found no significant relationship between reading scores and task-modulated connectivity. We also checked for an interaction between the PPI-reading association and group, observing a significant effect only in a small region in the left precentral and postcentral gyri (seeSupplementary Tables S3 and S4).

The distribution of out-of-scanner reading scores in our sample is shown inFigure 2b, separately for the seizure disorder and control groups. It is worth noting that while the distribution in the seizure disorder group (mean = 101, SD = 14) conforms to that expected from a normative population (i.e., mean of 100, standard deviation of 15;Wechsler, 2011), that of the control group is right shifted (mean = 109, SD = 10) with an under-representation of low reading scores (see Discussion 4.3).

## 4 Discussion

As hypothesized, our data show that the output of a single visual form processing region (FusG) is amplified along distinct connections depending on behavioural requirements. During linguistic processing of visual input, there is up-regulation of information flow between FusG and left perisylvian language cortex (Figs. 1dand3) relative to purely visuospatial processing. Conversely, during visuospatial judgments the same region in FusG shows increased connection with right parietal and right posterior middle temporal cortex, areas implicated in visuospatial processing (Fig. 1fand3). These task-dependent modulations of FusG connectivity were common to the control and seizure disorder groups. The seizure group included individuals with a wider spectrum of reading abilities. Among them, we observed greater increases in FusG connectivity to left middle frontal gyrus and medial prefrontal cortex in better readers, suggesting that visual text processing and articulatory sequencing are less tightly integrated in poor readers. Dynamic reconfiguration of networks can therefore be beneficial for cognitive performance.

**Fig. 3.**
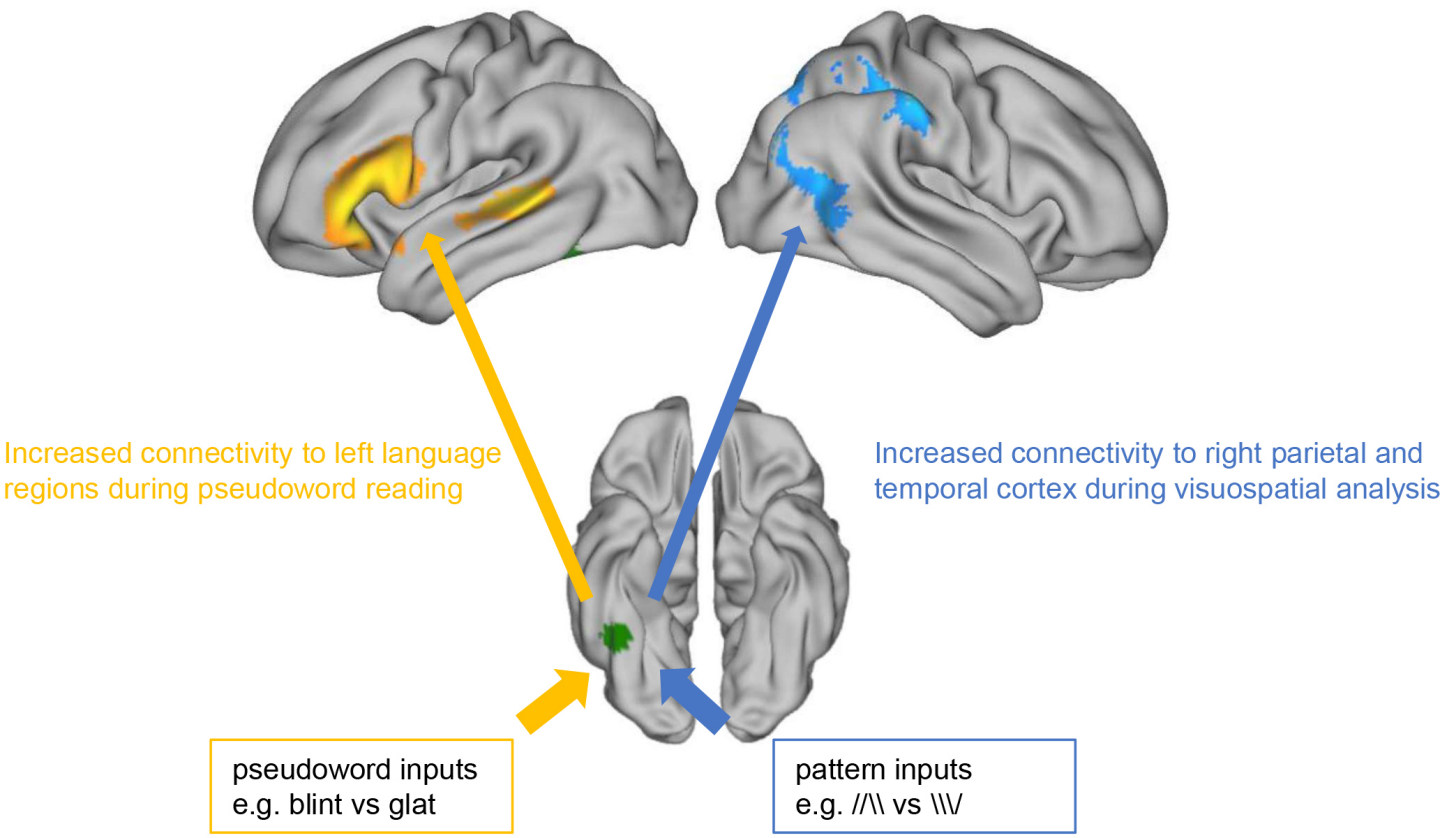
Illustrating our findings: During pseudoword reading, there is amplification of information flow between FusG and language cortex. Conversely, during visuospatial analysis, information flow from FusG switches to right parietal cortex. FusG seed location is shown in green. Left hemisphere on the left side.

### 4.1 Increased connectivity from left FusG to language and meta-language regions during pseudoword rhyming

Connectivity from left FusG to left language regions including left IFG and left STG increased during pseudoword rhyming, compared to visuospatial processing (Fig. 1d). In the context of nonword reading as used here, left IFG plays an important role in formulating an articulatory code for sounding out previously unencountered nonword letter strings (Flinker et al., 2015;Price, 2012). It integrates closely with left STG forming a “resonant circuit”, feeding back on one another to support active manipulation of phonological representations (Hickok & Poeppel, 2007;Sandak et al., 2004). Our data suggest that when the input to the language system is via text (graphemes), FusG plays a key role in establishing this dynamically realized circuit (Hickok & Poeppel, 2007;Sandak et al., 2004).

In addition to perisylvian language regions, we found positive task-modulated connectivity between FusG and right cerebellum (Crus I and II;Fig. 1d). Cerebellum is involved in motor planning and language processing (Marvel & Desmond, 2010), indexing the degree to which an intended movement deviates from the intention and providing corrective signals. This would be implicated in sounding out novel pseudowords, where participants must articulate a never before encountered letter string (Alvarez & Fiez, 2018;Marvel & Desmond, 2010;Nakatani et al., 2023). Our data suggest that the FusG output in response to text is not only tightly coupled with phonological processing mechanisms in the perisylvian cortex, but also interacts with mechanisms in the cerebellum that monitor the execution of action planning during covert articulation.

We also observed positive task-modulated connectivity from FusG to the medial aspect of left SFG (Fig. 1d).Dosenbach et al. (2006), using a graph-based approach to brain network organization, proposed that superior medial frontal cortex is crucial for maintaining cognitive set during task execution (Dosenbach et al., 2006). Hence, we postulate that this medial frontal region is involved in sustaining an appropriate task set, namely setting the brain state for grapheme-to-phoneme conversion upon the appearance of visual text. Such a meta-language role for this area is consistent with the lesion literature, with strokes here producing transcortical motor aphasia (dynamic aphasia) in which the initiative and impetus for spontaneous speech is lost (Alario et al., 2006;Crosson et al., 2018;Freedman et al., 1984).

The above findings suggest that during reading of nonwords, a complex circuit encompassing visual form processing, grapheme-phoneme conversion, somatomotor speech planning, and self-monitoring is implemented. This relatively extensive circuit can be effectively applied to visual processing of text in FusG. However, there are a few other PPI studies of reading with inconsistent findings that warrant consideration.Cao et al. (2014)studied 15 Chinese-English bilinguals who performed an English pseudoword rhyming task. They did not observe any PPI effect from FusG to language areas, though their small sample size likely limited the capacity to detect such an effect. PPI studies are known to suffer from relatively low power (O’Reilly et al., 2012), often requiring larger samples to reveal group effects. It has been suggested that a sample size of at least 50 is required to achieve 80% sensitivity (Masharipov et al., 2024), which is met in our analysis with over 200 participants.Chen et al. (2019)considered PPI effects in two separate data sets seeded from FusG during a real word rhyming task (*n*= 16 participants) and during a visual flanker task (*n*= 26). In both PPI analyses comparing against a resting baseline, the authors reported significant PPI effects from FusG to both language and attentional regions (Chen et al., 2019). A strength of our work is that we directly contrasted pseudoword rhyming with visuospatial pattern matching, aligning states as much as possible but for linguistic demands.

### 4.2 Coupling of left FusG with right attentional systems increases during visuospatial processing

To the best of our knowledge, up-regulation of connectivity from FusG during visuospatial processing relative to language processing has not been investigated. In our analysis, we found robust evidence of heightened connection from FusG to right intraparietal sulcus and adjacent cortex, right posterior temporal cortex, and to cortex near the intersection of the right MFG and precentral gyrus (frontal eye field) during pattern matching relative to pseudoword rhyming (Fig. 1f). These right hemisphere regions are strongly implicated in attentional and visuospatial processing (Chen et al., 2019;Husain & Nachev, 2007;Krauzlis, 2013), with right posterior parietal cortex and adjacent parieto-occipital regions implicated in judgments of line orientation specifically (Tranel et al., 2009). Our data imply that during the visuospatial processing demanded by the pattern matching task used here, the analysis of basic visual form in FusG becomes tightly coupled with visuospatial attentional systems in this right frontoparietal network.

### 4.3 FusG integrates more tightly with articulation and state setting systems in better readers

In our sample, the seizure disorder group encompassed a greater spread of reading abilities, including increased sampling of weaker readers. Within this group we observed that task-modulated connectivity from FusG was associated with reading proficiency. Specifically, we found a positive linear relationship between reading ability and increased FusG connectivity to left MFG, extending posteriorly into pre- and postcentral gyri (seeFig. 2a). This territory—recently referred to as middle precentral gyrus (midPrCG;Silva et al., 2022)—is dorsal to classical Broca’s area, proximal to premotor areas, and is thought to be involved in the phonological-motoric aspects of speech planning, especially the sequencing and articulation of novel syllabic sequences (Price, 2012;Silva et al., 2022). This aspect is particularly important for the decoding of pseudowords, which are by definition compromised of novel combinations of letters. Our data suggest that among more skilled readers, the outputs of graphemic processing in FusG are more amplified along connections to the speech sequencing and planning regions in left MFG, yielding a more tightly integrated processing network. Also in the seizure group, we found positive relationships between reading ability and FusG connectivity in the bilateral medial prefrontal cortex. These regions have been implicated in cognitive resource allocation during demanding tasks (Carter et al., 1998;Duncan & Owen, 2000;Fornito et al., 2004;Gennari et al., 2018), suggesting that proficient readers in the seizure group are able to engage and sustain a “reading set” in response to the rhyming task blocks.

We did not see these same reading ability-connectivity relationships in the control group. Given that both the seizure and control groups achieved near perfect scores on the baseline visuospatial pattern matching task, it is unlikely that task engagement confounded the results. A potential limiting factor is the comparatively restricted and rightward shifted distribution of reading scores in our control group, with an underrepresentation of individuals with low reading scores (Fig. 2b). We note that the relationships with reading scores in left MFG and bilateral prefrontal regions were not statistically different between the seizure and control groups, and it is possible that this effect plateaus as people become proficient readers. By this account, the lack of a significant relationship in controls would follow from most of the sample falling in the plateau range. Another potential account is that comparable behavioural performance levels between the seizure group and controls may nonetheless be attained via different underlying processing requirements, with good readers among the seizure group engaging more of an overt, effortful FusG to left premotor circuit to covertly sound out the nonwords, while skilled reading among the control group occurs in a more automatic, overlearned manner less reliant upon this phonological-motoric circuit. This was mentioned inWaites et al. (2006), where controls were found to have higher resting-state connectivity among major reading nodes than temporal lobe epilepsy patients (Waites et al., 2006). This automaticity of network shaping in support of reading may relate to the elevated rate of reading difficulties seen in epilepsy more generally (Lah et al., 2017;Tailby et al., 2014;Waites et al., 2006). A control group with a broader distribution of reading scores would help differentiate these potential explanations.

### 4.4 Multiplexing as a general principle of cortical processing

Previous research showed anatomical and functional connectivity in the reading system, specifically between FusG, STG, and IFG (e.g.,Chen et al., 2019). There is also research showing that the FusG is structurally and functionally connected to certain regions in the attentional network (Chen et al., 2019). Our findings thus add to a developing literature supporting a multiplex model of FusG (Chen et al., 2019;Price & Devlin, 2003;Vogel et al., 2014), whereby this region is recruited into different processing systems as required to carry out different tasks. This multiplexing idea is closely associated with the neuronal recycling hypothesis (Dehaene & Cohen, 2007), which posits that brain regions are repurposed for different functions, and newly developed skills build upon existing neural circuits. It also aligns with the flexible hub theory (Cole et al., 2013) and plays a role in cognitive control (Cole, 2017;Ito et al., 2022). One interpretation of the multiplex role is that for a region such as FusG, there are a range of communication pathways available for the distribution of its outputs, and that there is selective amplification of this output along certain channels according to the behavioural needs of the organism. This selective gain of signal transfer could potentially be regulated by a bottom-up process triggered by initial analyses of the retinal image, or it could also be a top-down process, perhaps mediated via medial prefrontal structures. These ideas will need to be tested in future studies.

Finally, it is possible that the FusG connection flexibility we observed extends beyond reading and serves as a fundamental principle underpinning cognition. The human brain is remarkably versatile, capable of supporting a diverse range of functions from coordinating physical movements to making complex decisions (Cole et al., 2013;Park & Friston, 2013). This broad functional repertoire is achieved upon a relatively fixed underlying structure. Consequently, cognition and behavior may arise from dynamic functional interactions on a fixed structural connectivity, implying a one-to-many relationship between structure and function (Cole et al., 2013;Dodel et al., 2005;Friston et al., 1997;Hagoort, 2014;Park & Friston, 2013;Pessoa, 2019; seeMcIntosh, 2000;Stephan, 2004for reviews).

### 4.5 Limitations and future studies

The current work aimed to investigate task-dependent connectivity modulation in individuals spanning a wide range of reading abilities. Investigation of other attributes that might associate with reading ability is beyond the scope of the current research. We note that years of education and IQ are highly correlated with reading scores, hence we did not regress out these variables in the behavioural regression analysis. The Australian Epilepsy Project is ongoing, and we plan to address epilepsy-specific questions in future research.

### 4.6 Conclusion

When visual input is analysed for language-related content, the connection from FusG to left perisylvian language cortex and other language-related areas is amplified. However, when visual input is processed purely for visuospatial form, the connection from FusG to these language related areas is downregulated, while connections from FusG to right hemisphere attentional systems are amplified. Our findings highlight the multiplexing role of FusG, where its outputs can be enhanced through specific connections based on behavioral demands. This dynamic reconfiguration of functional brain networks is beneficial for cognitive performance.

## Supplementary Material

Supplementary Material

## Data Availability

Any requests for access to the data used in this project should be directed to the Australian Epilepsy Project (a formal data access request can be lodged athttps://www.epilepsyproject.org.au/research/access-to-aep-data). The analyses were conducted using publicly available software, and no custom code was developed other than a modification described in the manuscript.
