# Supplementary material for: Task demands shape network interactions during reading and visual form processing
Source: Imaging Neurosci (Camb). 2025 May 28;3:IMAG.a.13. doi: 10.1162/IMAG.a.13 (PMC12319764; doi:10.1162/IMAG.a.13)
Supplement: Supplementary Material [file imag.a.13_supp.pdf]

Supplementary Information

Task demands shape network interactions during reading and visual form processing

Vicky He*[1,2], Bahman Tahayori[1,2], David N. Vaughan[1,2,3], Heath R. Pardoe[1,2], Jodie E. Chapman[1], Graeme D. Jackson[1,2,3], David F. Abbott[†,*1,2,4], and Chris Tailby[†,*1,2,5], for the Australian Epilepsy Project Investigators

[1]The Florey Institute of Neuroscience and Mental Health, Heidelberg, Victoria, Australia

[2]Florey Department of Neuroscience and Mental Health, The University of Melbourne, Parkville, Victoria, Australia

[3]Department of Neurology, Austin Health, Heidelberg, Victoria, Australia

[4]Department of Medicine - Austin Health, The University of Melbourne, Heidelberg, Victoria, Australia

[5]Department of Clinical Neuropsychology, Austin Health, Heidelberg, Victoria, Australia

[†]Joint senior authors

*Email for correspondence: he.v@unimelb.edu.au; david.abbott@florey.edu.au; chris.tailby@florey.edu.au

List of Contents

Supplementary Figure 1

Supplementary Tables 1 - 5

Supplementary Methods

Supplementary Figure 1

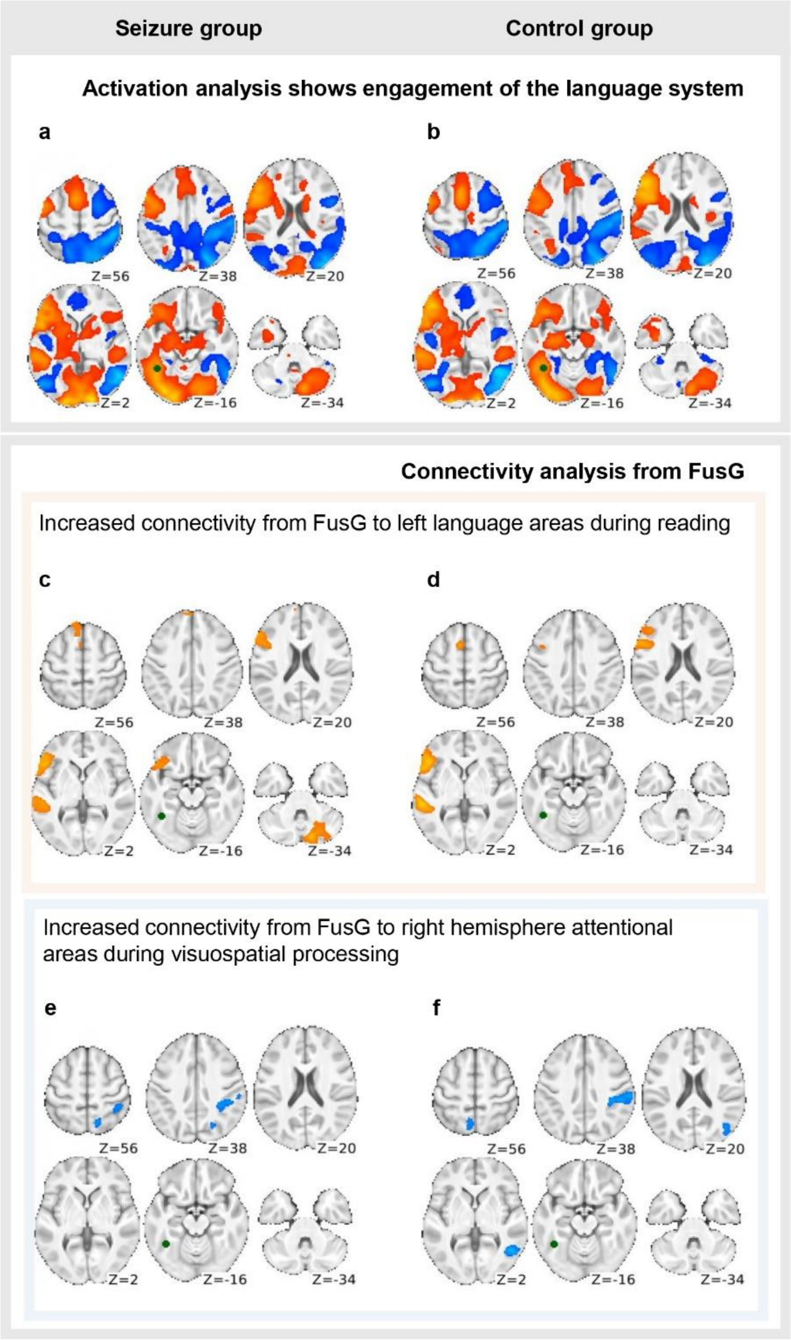

Fig. 1. Group specific pseudoword rhyming activation and FusG task-modulated connectivity analyses. a-b: One sample *t*-test showing activation (hot colours) and deactivation (cool colours) in pseudoword rhyming in the seizure group (panel a) and in the control group (panel b). c-d: One sample *t*-test showing regions that increase connectivity with FusG in pseudoword rhyming

in the seizure group (panel c) and in the control group (panel d). e-f: One sample *t*-tests showing regions that increase connectivity with FusG in visuospatial pattern matching in the seizure group (panel e) and in the control group (panel f). FusG seed location is shown in green (slice Z = -16). Left hemisphere on the left side. FWEc $p < 0.05$ (two-tailed).

Supplementary Tables 1 - 5

Table 1. Cluster details of regions that show increased connectivity from FusG in pseudoword rhyming at the combined group level.

	MNI			Cluster size
Left IFG (pars opercularis, pars triangularis), middle frontal gyrus (MFG), frontal pole, frontal orbital cortex	-48	26	-2	4820
Left STG	-54	-34	2	
	-56	20	10	
Right cerebellum (encompassing mainly Crus I and II, but also VI, VIIIa, VIIIb, IX)	36	-64	-26	2057
	28	-68	-24	
	20	-78	-44	
Left superior frontal gyrus (SFG); frontal pole, supplementary motor area (SMA), also precentral gyrus	-4	10	60	1528
	-8	24	60	
	-8	36	50	
Right occipital pole and lingual gyrus	20	-94	0	490
Left pre- and postcentral gyri, MFG	-54	-4	48	371

Table 2. Cluster details of regions that show increased connectivity from FusG in visuospatial pattern matching at the combined group level.

	MNI			Cluster size
Right intraparietal sulcus, supramarginal gyrus, postcentral gyrus, superior parietal lobule	60	-26	42	3450
	44	-34	38	
	16	-74	48	
Right precentral gyrus, SFG, MFG, frontal eye field	26	-6	50	723
	28	6	54	
	40	-2	38	
Left lateral occipital, precuneous	-10	-66	56	615
	-30	-44	50	
	-14	-74	48	
Left lateral occipital	-42	-70	12	468
	-38	-82	26	

Table 3. Cluster details of positive relationships between task-modulated connectivity from FusG and reading scores in the seizure group.

	MNI			Cluster Size
Bilateral anterior cingulate, paracingulate	14	34	36	1498
	-12	26	36	
	12	14	34	
Left MFG, precentral gyrus, IFG	-44	10	36	1089
	-46	2	40	
	-46	-4	34	
Left precentral gyrus, left postcentral gyrus, lateral	-34	-34	46	591
	-34	-22	52	
	-38	-14	56	
Left precentral gyrus, left postcentral gyrus, medial	-20	-22	70	422
	-12	-32	54	
	-18	-24	58	

Table 4. Cluster details of the reading score by group interaction effect.

	MNI			Cluster size
Left precentral gyrus, left postcentral gyrus, medial	-32	-34	48	359
	-10	-32	58	
	-14	-38	64	

Table 5. AEP investigator list with Contributor Roles Taxonomy (CRediT) author statement relevant for this manuscript.

Australian Epilepsy Project Investigators			
**Name & ORCID**	**Primary Location**	**Role**	**CRediT**
**Graeme D. Jackson, MD 0000-0002-7917-5326**	The Florey Institute of Neuroscience and Mental Health	Chief Investigator	Conceptualisation, Methodology, Investigation, Resources, Writing - Review & Editing, Supervision, Project Administration, Funding Acquisition
**David F. Abbott, PhD 0000-0002-7259-8238**	The Florey Institute of Neuroscience and Mental Health	Informatics Lead	Conceptualisation, Methodology, Software, Investigation, Resources, Data Curation, Writing - Original Draft, Writing - Review & Editing, Visualisation, Supervision, Project Administration, Funding Acquisition

**Zanfina Ademi, PhD 0000-0002-0625-3522**	Monash University	Health Economics Lead	Conceptualisation, Funding acquisition
**Subhaga Amarasekara**	The Florey Institute of Neuroscience and Mental Health	Product Lead	Resources, Project Administration
**Amanda Anderson**	The Florey Institute of Neuroscience and Mental Health	Lived Experience Ambassador and Participant Lead	Investigation, Resources, Funding acquisition
**Rachel Hughes**	The Florey Institute of Neuroscience and Mental Health	Clinical Research Coordinator	Investigation, Resources
**Donna Hutchison**	The Florey Institute of Neuroscience and Mental Health	Executive Lead	Project administration
**Patrick Kwan, MD 0000-0001-7310-276X**	Monash University	Outcomes Lead	Conceptualisation, Resources, Funding acquisition
**Paul Lightfoot**	The Florey Institute of Neuroscience and Mental Health	Operations Lead	Investigation, Project administration
**Saul Mullen, MD, PhD 0000-0003-1224-4101**	The University of Melbourne	Protocol Development Lead (2019-2021)	Conceptualisation, Methodology, Funding acquisition

**Karen L. Oliver, PhD 0000-0001-5188-6153**	The University of Melbourne	Genetics Lead	Conceptualisation, Funding acquisition
**Heath R. Pardoe, PhD 0000-0002-0123-2167**	The Florey Institute of Neuroscience and Mental Health	Science Operations Lead	Investigation, Resources, Writing - Review & Editing, Project Administration
**Mangor Pedersen, PhD 0000-0002-9199-1916**	Auckland University of Technology	Artificial Intelligence Lead	Conceptualisation, Methodology, Funding acquisition
**Chris Tailby, PhD 0000-0002-1320-5924**	The Florey Institute of Neuroscience and Mental Health	Neuropsychology Lead	Conceptualisation, Methodology, Investigation, Resources, Data Curation, Writing - Original Draft, Writing - Review & Editing, Visualisation, Supervision, Project Administration, Funding Acquisition

**David N. Vaughan, MD, PhD 0000-0002-6225-7739**	The Florey Institute of Neuroscience and Mental Health	Imaging Lead	Conceptualisation, Methodology, Investigation, Resources, Writing - Review & Editing, Project Administration, Funding Acquisition
**Anton De Weger 0009-0006-7478-361X**	The Florey Institute of Neuroscience and Mental Health	Digital and Technology Lead	Software, Resources, Data Curation

Supplementary Methods

Marchenko-Pastur PCA (MPPCA) de-noising (Veraart et al. 2016) was performed using the dwidenoise command from MRtrix3 software version 3.0.4 (Tournier et al. 2019), with window size 7 × 7 × 7 using double precision calculations.

For each subject, results included in this manuscript come from preprocessing performed using *fMRIPrep* 21.0.2 (Esteban, Markiewicz, et al. (2018); Esteban, Blair, et al. (2018); RRID:SCR_016216), which is based on *Nipype* 1.6.1 (K. Gorgolewski et al. (2011); K. J. Gorgolewski et al. (2018); RRID:SCR_002502).

Preprocessing of B_0 inhomogeneity mappings

A total of 2 fieldmaps were found available within the input BIDS structure for this particular subject. A B_0-nonuniformity map (or *fieldmap*) was estimated based on two (or more) echo-planar imaging (EPI) references with topup (Andersson, Skare, and Ashburner (2003); FSL 6.0.5.1:57b01774).

Anatomical data preprocessing

A total of 1 T1-weighted (T1w) images were found within the input BIDS dataset.The T1-weighted (T1w) image was corrected for intensity non-uniformity (INU) with N4BiasFieldCorrection (Tustison et al. 2010), distributed with ANTs 2.3.3 (Avants et al. 2008, RRID:SCR_004757), and used as T1w-reference throughout the workflow. The T1w-reference was then skull-stripped with a *Nipype* implementation of the antsBrainExtraction.sh workflow (from ANTs), using OASIS30ANTs as target template. Brain tissue segmentation of cerebrospinal fluid (CSF), white-matter (WM) and gray-matter (GM) was performed on the brain-extracted T1w using fast (FSL 6.0.5.1:57b01774, RRID:SCR_002823, Zhang, Brady, and Smith 2001). Brain surfaces were reconstructed using recon-all (FreeSurfer 6.0.1, RRID:SCR_001847,

Dale, Fischl, and Sereno 1999), and the brain mask estimated previously was refined with a custom variation of the method to reconcile ANTs-derived and FreeSurfer-derived segmentations of the cortical gray-matter of Mindboggle (RRID:SCR_002438, Klein et al. 2017). Volume-based spatial normalization to two standard spaces (MNI152NLin6Asym, MNI152NLin2009cAsym) was performed through nonlinear registration with antsRegistration (ANTs 2.3.3), using brain-extracted versions of both T1w reference and the T1w template. The following templates were selected for spatial normalization: *FSL's MNI ICBM 152 non-linear 6th Generation Asymmetric Average Brain Stereotaxic Registration Model* [Evans et al. (2012), RRID:SCR_002823; TemplateFlow ID: MNI152NLin6Asym], *ICBM 152 Nonlinear Asymmetrical template version 2009c* [Fonov et al. (2009), RRID:SCR_008796; TemplateFlow ID: MNI152NLin2009cAsym].

Functional data preprocessing

For each of the 1 BOLD runs found per subject (across all tasks and sessions), the following preprocessing was performed. First, a reference volume and its skull-stripped version were generated from the shortest echo of the BOLD run using a custom methodology of *fMRIPrep*. Head-motion parameters with respect to the BOLD reference (transformation matrices, and six corresponding rotation and translation parameters) are estimated before any spatiotemporal filtering using mcflirt (FSL 6.0.5.1:57b01774, Jenkinson et al. 2002). The estimated *fieldmap* was then aligned with rigid-registration to the target EPI (echo-planar imaging) reference run. The field coefficients were mapped on to the reference EPI using the transform. BOLD runs were slice-time corrected to 0.4s (0.5 of slice acquisition range 0s-0.8s) using 3dTshift from AFNI (Cox and Hyde 1997, RRID:SCR_005927). A $T2^\star$ map was estimated from the preprocessed EPI echoes, by voxel-wise fitting the maximal number of echoes with reliable signal in that voxel to a monoexponential signal decay model with nonlinear regression. The $T2^\star/S_0$ estimates from a log-linear regression fit were used for initial values. The calculated $T2^\star$

map was then used to optimally combine preprocessed BOLD across echoes following the method described in (Posse et al. 1999). The optimally combined time series was carried forward as the *preprocessed BOLD*. The BOLD reference was then co-registered to the T1w reference using bbregister (FreeSurfer) which implements boundary-based registration (Greve and Fischl 2009). Co-registration was configured with six degrees of freedom. First, a reference volume and its skull-stripped version were generated using a custom methodology of *fMRIPrep*. Several confounding time-series were calculated based on the *preprocessed BOLD*: framewise displacement (FD), DVARS and three region-wise global signals. FD was computed using two formulations following Power (absolute sum of relative motions, Power et al. (2014)) and Jenkinson (relative root mean square displacement between affines, Jenkinson et al. (2002)). FD and DVARS are calculated for each functional run, both using their implementations in *Nipype* (following the definitions by Power et al. 2014). The three global signals are extracted within the CSF, the WM, and the whole-brain masks. Additionally, a set of physiological regressors were extracted to allow for component-based noise correction (*CompCor*, Behzadi et al. 2007). Principal components are estimated after high-pass filtering the *preprocessed BOLD* time-series (using a discrete cosine filter with 128s cut-off) for the two *CompCor* variants: temporal (tCompCor) and anatomical (aCompCor). tCompCor components are then calculated from the top 2% variable voxels within the brain mask. For aCompCor, three probabilistic masks (CSF, WM and combined CSF+WM) are generated in anatomical space. The implementation differs from that of Behzadi et al. in that instead of eroding the masks by 2 pixels on BOLD space, the aCompCor masks are subtracted a mask of pixels that likely contain a volume fraction of GM. This mask is obtained by dilating a GM mask extracted from the FreeSurfer's *aseg* segmentation, and it ensures components are not extracted from voxels containing a minimal fraction of GM. Finally, these masks are resampled into BOLD space and binarized by thresholding at 0.99 (as in the original implementation). Components are also calculated separately within the WM and CSF masks. For each CompCor decomposition, the *k*

components with the largest singular values are retained, such that the retained components' time series are sufficient to explain 50 percent of variance across the nuisance mask (CSF, WM, combined, or temporal). The remaining components are dropped from consideration. The head-motion estimates calculated in the correction step were also placed within the corresponding confounds file. The confound time series derived from head motion estimates and global signals were expanded with the inclusion of temporal derivatives and quadratic terms for each (Satterthwaite et al. 2013). Frames that exceeded a threshold of 0.5 mm FD or 1.5 standardised DVARS were annotated as motion outliers. The BOLD time-series were resampled into standard space, generating a *preprocessed BOLD run in MNI152NLin6Asym space*. First, a reference volume and its skull-stripped version were generated using a custom methodology of *fMRIPrep*. The BOLD time-series were resampled onto the following surfaces (FreeSurfer reconstruction nomenclature): *fsaverage5*. All resamplings can be performed with *a single interpolation step* by composing all the pertinent transformations (i.e. head-motion transform matrices, susceptibility distortion correction when available, and co-registrations to anatomical and output spaces). Gridded (volumetric) resamplings were performed using antsApplyTransforms (ANTs), configured with Lanczos interpolation to minimize the smoothing effects of other kernels (Lanczos 1964). Non-gridded (surface) resamplings were performed using mri_vol2surf (FreeSurfer).

Many internal operations of *fMRIPrep* use *Nilearn* 0.8.1 (Abraham et al. 2014, RRID:SCR_001362), mostly within the functional processing workflow. For more details of the pipeline, see the section corresponding to workflows in *fMRIPrep*'s documentation.

Copyright Waiver

The above boilerplate text was automatically generated by fMRIPrep with the express intention that users should copy and paste this text into their manuscripts *unchanged*. It is released under the CC0 license.

Additional References

Abraham, Alexandre, Fabian Pedregosa, Michael Eickenberg, Philippe Gervais, Andreas Mueller, Jean Kossaifi, Alexandre Gramfort, Bertrand Thirion, and Gael Varoquaux. 2014. “Machine Learning for Neuroimaging with Scikit-Learn.” *Frontiers in Neuroinformatics* 8. https://doi.org/10.3389/fninf.2014.00014.

Andersson, Jesper L. R., Stefan Skare, and John Ashburner. 2003. “How to Correct Susceptibility Distortions in Spin-Echo Echo-Planar Images: Application to Diffusion Tensor Imaging.” *NeuroImage* 20 (2): 870–88. https://doi.org/10.1016/S1053-8119(03)00336-7.

Avants, B. B., C. L. Epstein, M. Grossman, and J. C. Gee. 2008. “Symmetric Diffeomorphic Image Registration with Cross-Correlation: Evaluating Automated Labeling of Elderly and Neurodegenerative Brain.” *Medical Image Analysis* 12 (1): 26–41. https://doi.org/10.1016/j.media.2007.06.004.

Behzadi, Yashar, Khaled Restom, Joy Liau, and Thomas T. Liu. 2007. “A Component Based Noise Correction Method (CompCor) for BOLD and Perfusion Based fMRI.” *NeuroImage* 37 (1): 90–101. https://doi.org/10.1016/j.neuroimage.2007.04.042.

Cox, Robert W., and James S. Hyde. 1997. “Software Tools for Analysis and Visualization of fMRI Data.” *NMR in Biomedicine* 10 (4-5): 171–78. https://doi.org/10.1002/(SICI)1099-1492(199706/08)10:4/5<171::AID-NBM453>3.0.CO;2-L.

Dale, Anders M., Bruce Fischl, and Martin I. Sereno. 1999. “Cortical Surface-Based Analysis: I. Segmentation and Surface Reconstruction.” *NeuroImage* 9 (2): 179–94. https://doi.org/10.1006/nimg.1998.0395.

Esteban, Oscar, Ross Blair, Christopher J. Markiewicz, Shoshana L. Berleant, Craig Moodie, Feilong Ma, Ayse Ilkay Isik, et al. 2018. “fMRIPrep.” *Software*. https://doi.org/10.5281/zenodo.852659.

Esteban, Oscar, Christopher Markiewicz, Ross W Blair, Craig Moodie, Ayse Ilkay Isik, Asier

Erramuzpe Aliaga, James Kent, et al. 2018. “fMRIPrep: A Robust Preprocessing Pipeline for Functional MRI.” *Nature Methods*. https://doi.org/10.1038/s41592-018-0235-4.

Evans, AC, AL Janke, DL Collins, and S Baillet. 2012. “Brain Templates and Atlases.” *NeuroImage* 62 (2): 911–22. https://doi.org/10.1016/j.neuroimage.2012.01.024.

Fonov, VS, AC Evans, RC McKinstry, CR Almli, and DL Collins. 2009. “Unbiased Nonlinear Average Age-Appropriate Brain Templates from Birth to Adulthood.” *NeuroImage* 47, Supplement 1: S102. https://doi.org/10.1016/S1053-8119(09)70884-5.

Gorgolewski, K., C. D. Burns, C. Madison, D. Clark, Y. O. Halchenko, M. L. Waskom, and S. Ghosh. 2011. “Nipype: A Flexible, Lightweight and Extensible Neuroimaging Data Processing Framework in Python.” *Frontiers in Neuroinformatics* 5: 13. https://doi.org/10.3389/fninf.2011.00013.

Gorgolewski, Krzysztof J., Oscar Esteban, Christopher J. Markiewicz, Erik Ziegler, David Gage Ellis, Michael Philipp Notter, Dorota Jarecka, et al. 2018. “Nipype.” *Software*. https://doi.org/10.5281/zenodo.596855.

Greve, Douglas N, and Bruce Fischl. 2009. “Accurate and Robust Brain Image Alignment Using Boundary-Based Registration.” *NeuroImage* 48 (1): 63–72. https://doi.org/10.1016/j.neuroimage.2009.06.060.

Jenkinson, Mark, Peter Bannister, Michael Brady, and Stephen Smith. 2002. “Improved Optimization for the Robust and Accurate Linear Registration and Motion Correction of Brain Images.” *NeuroImage* 17 (2): 825–41. https://doi.org/10.1006/nimg.2002.1132.

Klein, Arno, Satrajit S. Ghosh, Forrest S. Bao, Joachim Giard, Yrjö Häme, Eliezer Stavsky, Noah Lee, et al. 2017. “Mindboggling Morphometry of Human Brains.” *PLOS Computational Biology* 13 (2): e1005350. https://doi.org/10.1371/journal.pcbi.1005350.

Lanczos, C. 1964. “Evaluation of Noisy Data.” *Journal of the Society for Industrial and Applied Mathematics Series B Numerical Analysis* 1 (1): 76–85. https://doi.org/10.1137/0701007.

Posse, Stefan, Stefan Wiese, Daniel Gembris, Klaus Mathiak, Christoph Kessler, Maria-Liisa

Grosse-Ruyken, Barbara Elghahwagi, Todd Richards, Stephen R. Dager, and Valerij G. Kiselev. 1999. “Enhancement of BOLD-Contrast Sensitivity by Single-Shot Multi-Echo Functional MR Imaging.” *Magnetic Resonance in Medicine* 42 (1): 87–97. https://doi.org/10.1002/(SICI)1522-2594(199907)42:1<87::AID-MRM13>3.0.CO;2-O.

Power, Jonathan D., Anish Mitra, Timothy O. Laumann, Abraham Z. Snyder, Bradley L. Schlaggar, and Steven E. Petersen. 2014. “Methods to Detect, Characterize, and Remove Motion Artifact in Resting State fMRI.” *NeuroImage* 84 (Supplement C): 320–41. https://doi.org/10.1016/j.neuroimage.2013.08.048.

Satterthwaite, Theodore D., Mark A. Elliott, Raphael T. Gerraty, Kosha Ruparel, James Loughead, Monica E. Calkins, Simon B. Eickhoff, et al. 2013. “An improved framework for confound regression and filtering for control of motion artifact in the preprocessing of resting-state functional connectivity data.” *NeuroImage* 64 (1): 240–56. https://doi.org/10.1016/j.neuroimage.2012.08.052.

Tustison, N. J., B. B. Avants, P. A. Cook, Y. Zheng, A. Egan, P. A. Yushkevich, and J. C. Gee. 2010. “N4itk: Improved N3 Bias Correction.” *IEEE Transactions on Medical Imaging* 29 (6): 1310–20. https://doi.org/10.1109/TMI.2010.2046908.

Zhang, Y., M. Brady, and S. Smith. 2001. “Segmentation of Brain MR Images Through a Hidden Markov Random Field Model and the Expectation-Maximization Algorithm.” *IEEE Transactions on Medical Imaging* 20 (1): 45–57. https://doi.org/10.1109/42.906424.
